# The chromatin remodeler DDM1 promotes hybrid vigor by regulating salicylic acid metabolism

**DOI:** 10.1038/celldisc.2016.27

**Published:** 2016-08-09

**Authors:** Qingzhu Zhang, Yanqiang Li, Tao Xu, Ashish Kumar Srivastava, Dong Wang, Liang Zeng, Lan Yang, Li He, Heng Zhang, Zhimin Zheng, Dong-Lei Yang, Cheng Zhao, Juan Dong, Zhizhong Gong, Renyi Liu, Jian-Kang Zhu

**Affiliations:** 1 Shanghai Centre for Plant Stress Biology, Shanghai Institutes for Biological Sciences, Chinese Academy of Sciences, Shanghai, China; 2 Shanghai Institute of Plant Physiology and Ecology, Shanghai Institutes of Biological Sciences, Chinese Academy of Sciences, Shanghai, China; 3 Waksman Institute of Microbiology, Rutgers University, Piscataway, NJ, USA; 4 State Key Laboratory of Plant Physiology and Biochemistry, College of Biological Sciences, China Agricultural University, Beijing, China; 5 Department of Horticulture and Landscape Architecture, Purdue University, West Lafayette, IN, USA

**Keywords:** DDM1, DNA methylation, epigenetics, heterosis, non-additive expression

## Abstract

In plants, hybrid vigor is influenced by genetic and epigenetic mechanisms; however, the molecular pathways are poorly understood. We investigated the potential contributions of epigenetic regulators to heterosis in *Arabidposis* and found that the chromatin remodeler DECREASED DNA METHYLATION 1 (DDM1) affects early seedling growth heterosis in Col/C24 hybrids. *ddm1* mutants showed impaired heterosis and increased expression of non-additively expressed genes related to salicylic acid metabolism. Interestingly, our data suggest that salicylic acid is a hormetic regulator of seedling growth heterosis, and that hybrid vigor arises from crosses that produce optimal salicylic acid levels. Although DNA methylation failed to correlate with differential non-additively expressed gene expression, we uncovered DDM1 as an epigenetic link between salicylic acid metabolism and heterosis, and propose that the endogenous salicylic acid levels of parental plants can be used to predict the heterotic outcome. Salicylic acid protects plants from pathogens and abiotic stress. Thus, our findings suggest that stress-induced hormesis, which has been associated with increased longevity in other organisms, may underlie specific hybrid vigor traits.

## Introduction

Hybrid vigor, or heterosis, refers to the improved performance of hybrid offspring relative to their parents. This is often restricted to a particular trait such as yield, plant height, biomass or defense and can be influenced by parent background and imprinting [1]. Various mechanisms, including dominance, overdominance and epistasis, have been proposed to explain heterosis from a quantitative genetics perspective; however, the molecular mechanisms underlying heterosis are still unclear. Recently, heterosis was found to correlate with circadian-clock-mediated regulation of several biotic and abiotic stress-related genes [2]. An overdominant gene, *SINGLE FLOWER TRUSS*, has also been identified in tomato, which regulates fruit yield heterosis [3]. In addition, the role of polyploidy and epigenetic interactions between parents, including small RNA, DNA methylation and histone modifications, have also been proposed to influence heterosis [4–8]. Indeed, *Arabidopsis* accessions C24/Ler, which have very similar genomic sequences, show heterosis. The associated epigenetic modifications, including DNA cytosine methylation, have also shown segregation, which may contribute to a loss of heterosis in the F_2_ generation [9]. However, the key epigenetic mechanisms regulating heterosis have not been investigated using genetic tests.

RNA-directed DNA methylation (RdDM) is a well-studied epigenetic pathway in plants and has been implicated in heterosis due to the alteration of small RNA profiles after hybridizations [10–13], although there is also evidence that it is not important for heterosis [14, 15]. In the current model of RdDM, *de novo* DNA methylation is initiated by the production of 24-nt small interfering RNAs (siRNAs), which are generated by a set of factors, including a plant-specific RNA polymerase (Pol IV), RNA-dependent RNA polymerase 2 (RDR2) and Dicer-like 3. These 24-nt siRNAs are then loaded onto AGO4/6 to form an AGO4/6–siRNA complex. Guided by siRNAs, the AGO4/6–siRNA complex binds to nascent noncoding RNAs produced by another plant-specific RNA polymerase (Pol V), through RNA–RNA base pairing. Recent work suggests that the role of siRNAs can be largely replaced by their precursors, which are Pol IV and RDR2 products of 25–50 nt in length [16]. Pol V function also requires the DDR complex (consisting of DEFECTIVE IN RNA-DIRECTED DNA METHYLATION 1, DEFECTIVE IN MERISTEM SILENCING 3 and RNA-DIRECTED DNA METHYLATION 1) and formation of the AGO4/6–siRNA–scaffold RNA complex may require RNA-DIRECTED DNA METHYLATION 3. Eventually, DOMAINS REARRANGED METHYLTRANSFERASE 2 is recruited to this complex and mediates *de novo* methylation of cytosines in all sequence contexts (CG, CHG and CHH, where H represents A, C or T). This results in transcriptional silencing at genomic loci transcribed by Pol V. Overall, RdDM has been found to occur at a wide range of genomic locations but with preferences for euchromatic regions.

RdDM is excluded to some extent from pericentromeric heterochromatin regions surrounding centromeres [17]. DNA methylation in these regions mostly occurs through an siRNA-independent manner and relies on another SWI2/SNF2-like chromatin remodeler protein called DDM1 (DECREASED DNA METHYLATION 1) [18, 19]. In *Arabidopsis*, DDM1 preferentially affects DNA methylation in heterochromatin, transposable elements and tandem repeat-rich regions, and primarily targets long terminal repeat retrotransposons and Mutator DNA transposons. In addition, DDM1 can modulate the expression of protein-coding genes by regulating their adjacent transposable elements (TEs) [20]. Recent work also suggested that DDM1 functions separately from RdDM by allowing the DNA methyltransferase CHROMOMETHYLASE 2 to approach H1-containing heterochromatin [17]. In addition, DDM1 can regulate gene expression through histone modifications [21].

Here we used epigenetic mutants to investigate the early seedling biomass heterosis of *Arabidopsis* Col and C24 accessions. We uncovered DDM1 as a major regulator of heterosis. We found that genes associated with heterosis were generally not regulated by DNA methylation. Further, high-throughput transcriptomics revealed that DDM1 affects salicylic acid (SA)-related genes. SA defends plants from pathogen infection and abiotic stresses, such as drought [22]. Previous findings have suggested that decreased SA contributed to the increased growth of hybrid plants [8, 23]. However, our findings suggest that SA is a hormetic regulator of biomass heterosis and that, depending on the parent, optimal levels of SA can be associated with improved hybrid growth. Thus, our findings indicate that DDM1 connects epigenetics, SA metabolism and hormesis, to the broader context of heterosis regulation in plants.

## Results

### Heterosis of F_1_ hybrids was impaired in *ddm1* loss-of-function mutants

**Figure 1:**
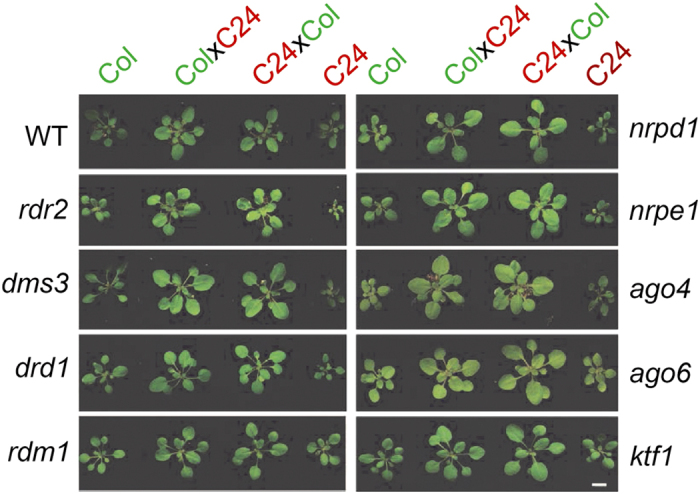
Heterotic phenotypes of F_1_ hybrids from Col and C24 crosses in wild-type and RdDM mutant backgrounds. F_1_ hybrids were produced from Col and C24 crosses using wild-type and RdDM pathway mutant plants. As a convention, the maternal parent is listed first. The mutant details are described in Supplementary Table S1. Seedlings from all genotypes were gown in pots and then phenotyping was conducted at 18–20 DAS (day after sowed) (scale bar=2 cm.) For quantitative assessment, refer Supplementary Figure S1J. The experiments were performed in triplicate to confirm reproducibility.

To test the roles of epigenetic regulators in heterosis, we used a series of homozygous mutants in the C24 background [24–26] and crossed them with their corresponding homozygous alleles in the Col ecotype (for gene accession numbers and SALK IDs of Col and C24, see Supplementary Figure S1A–I and Supplementary Table S1). We made reciprocal crosses between Col_maternal_ × C24_paternal_ and C24_maternal_×Col_paternal_ to obtain F1 and r-F1 hybrids, respectively, and confirmed that they were true hybrids by genotyping (Supplementary Figure S1). We found that F_1_ seedlings from wild type (WT), *rdr2*, *dms3*, *drd1*, *rdm1*, *nrpd1*, *nrpe1*, *ago4*, *ago6* and *rdm3* crosses displayed a significant increase in leaf width compared with their respective parents, indicating a heterosis phenotype (Figure 1). No significant difference in phenotype was observed between reciprocal crosses (Figure 1 and Supplementary Figure S1J).

**Figure 2:**
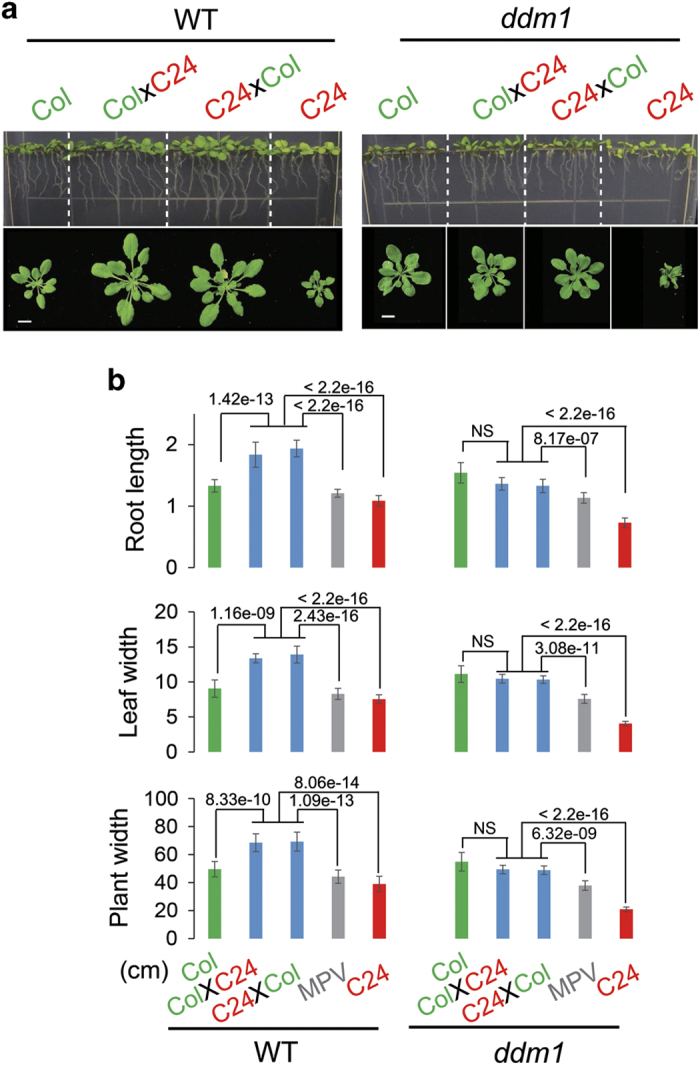
Heterosis was impaired in F_1_ offspring of Col and C24 in *ddm1* mutant background. The phenotyping of F1 wild-type and *ddm1* mutant plants was performed by the pot and vertical plate method. WT-F_1_ showed BPH, whereas *ddm1*-F_1_ showed MPH performance (**a**). The differential phenotype was quantified in terms of root length (at 10 DAS), leaf width and plant width (at 20 DAS) (**b**). The *P-*values are shown for corresponding pair-wise comparisons (NS, not significant; *P*-values>0.05, *t*-test). The *ddm1-10* (Col) and *ddm1-15* (C24) were used in this experiment. The experiments were performed in triplicate, to confirm reproducibility. MPV, mid-parent values (scale bar, 1 cm).

To determine whether the chromatin remodeler DDM1 affects heterosis, we generated F_1_ hybrids of *ddm1* loss-of-function mutants. We performed independent reciprocal crosses between three Col (*ddm1-1*, *ddm1-10* and *ddm1-16*) and three C24 (*ddm1-9*, *ddm1-14* and *ddm1-15*) *ddm1* alleles. The F_1_ hybrids from different allelic combinations were genotyped to confirm that they are true hybrids (Figure 2 and Supplementary Figure S2). We recently derived *ddm1-14*, *ddm1-15* and *ddm1-16*, and verified their effects by evaluating the methylation status of 5S-rDNA through methylation-sensitive restriction enzyme PCR (Chop-PCR) (Supplementary Figure S2H) [27]. As *ddm1* mutants show developmental defects after a few generations of inbreeding [28], homozygous *ddm1* plants were used for only three generations in heterosis tests and then fresh homozygous seeds were obtained from heterozygous parents.

We examined the growth pattern at the early seedling stage and found that heterosis was markedly impaired in *ddm1*-F_1_ hybrids compared with WT-F_1_ hybrids (Figure 2a). The key morphological traits of seedlings such as root length, leaf width and rosette width were quantified. The WT-F_1_ hybrids showed significantly better performance than both parents (*P*<0.01, *t*-test), confirming best-parent heterosis (BPH). Unlike WT-F_1_, *ddm1*-F_1_ hybrids showed a dramatic reduction in these morphological traits when compared with WT-F_1_ (Supplementary Figure S3D). Although *ddm1*-F_1_ plants displayed a growth pattern that was more vigorous than the parental *ddm1*-C24 plants, it was similar to parental *ddm1*-Col plants. Therefore, *ddm1*-F_1_ seedlings lost the BPH that was observed in WT-F_1_. However, their growth vigor was better than the average performance of the two parents, consistent with mid-parent heterosis (MPH) (Figure 2b). We also evaluated different alleles of *ddm1* and did not detect significant differences among the different allelic combinations (Supplementary Figure S3A–D).

To test whether mutations in both *DDM1* alleles are required for the impairment of hybrid vigor, we performed reciprocal crosses with a *ddm1* mutant in one ecotype and WT in the other ecotype, and examined the heterosis of F_1_ hybrids. Heterozygous F_1_ hybrids from all four crosses, including *ddm1*-Col×*DDM1*-C24, *ddm1*-C24×*DDM1*-Col, *DDM1*-Col×*ddm1*-C24 and *DDM1*-C24×*ddm1*-Col, were compared with their corresponding parents and with WT-F_1_ hybrids as well. No obvious reduction in early seedling growth heterosis was observed in these crosses (Supplementary Figure S3E and F), indicating that loss of BPH requires dysfunction of both *DDM1* alleles in F_1_ hybrids. Further, these results demonstrate that impaired heterosis in *ddm1*-Col and *ddm1*-C24 crosses is not simply due to the slower growth rate of *ddm1*-C24 seedlings.

### NEGs related to SA in F_1_ hybrids

To investigate the molecular basis of BPH-to-MPH conversion in *ddm1*-F_1_, we identified differentially expressed genes (DEGs) in different genotypes, but the DEGs failed to provide a clear explanation of the change in heterosis (Supplementary Information and Supplementary Figures S4 and S5).

**Figure 3:**
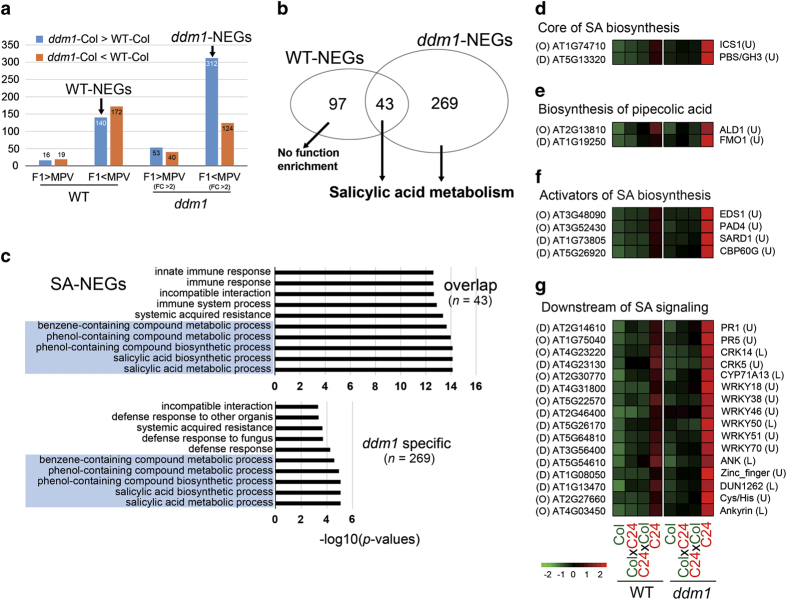
The functional NEGs were enriched in SA metabolism (SA-NEGs). Genes showing differential expression compared with MPVs were defined as NEGs in WT- and *ddm1*-F_1_ (**a**). Venn diagram shows the overlap of WT- and *ddm1*-NEGs and their functional enrichment in SA metabolism (**b**). GO-based functional analysis showed that overlapped NEGs between WT and *ddm1* are significantly enriched in SA metabolism (**c**). The NEGs related to SA metabolism were further classified on the basis of their involvement with core SA biosynthesis (**d**), biosynthesis of pipecolic acid (**e**), activator of SA biosynthesis (**f**) and downstream to SA signaling (**g**). The letter ‘O’ and ‘D’ in brackets before gene IDs indicated that the genes were from ‘overlap’ or ‘*ddm1* specifc’ groups in **c**, respectively. The letter ‘L’ and ‘U’ in brackets after gene names indicated that the gene were from ‘Low-methylation’ or ‘Unmethylated’ groups in Supplementary Figure S7.

We then analysed non-additively expressed genes (NEGs) in WT-F_1_ and *ddm1*-F_1_. NEGs are defined by F1 gene expression that is significantly different from the average value of the parental inbred lines (mid-parent value, MPV). We identified 347 NEGs in WT-F_1_, using *t*-test (two-sides) and *P*-value<0.05 as cutoff, of which 35 and 312 genes were above or below the MPV, respectively. Using the same criteria, along with a fold change of >2 cutoff to focus on highly functional NEGs, we identified 529 NEGs in *ddm1*-F_1_, of which 93 and 436 were above or below the MPV, respectively. The four classes of NEGs (above or below MPV in WT-F_1_ and *ddm1*-F_1_) were further divided into two groups depending on their upregulation (*ddm1*-Col>WT-Col) or downregulation (*ddm1*-Col<WT-Col) in the *ddm1* mutant (Figure 3a). This resulted in eight groups of NEGs, which were independently tested for gene ontology (GO)-based functional enrichment analysis. Only two of these groups displayed functional enrichments, which we subsequently refer to as WT-NEGs (140 genes) and *ddm1*-NEGs (312 genes) (Figure 3a).

Forty-three of the 140 WT-NEGs are related to SA metabolism and response, whereas the remaining WT-NEGs did not show any functional enrichment (Figure 3b). The full set of 312 *ddm1*-NEGs are statistically enriched in SA, including the 43 WT-NEGs related to SA (Figure 3b and c). For convenience, we refer to these 312 NEGs as SA-NEGs. We further classified the SA-NEGs based on their association with different roles in SA metabolism or response, including core components of SA biosynthesis, activators of SA biosynthesis, downstream of SA signaling and other genes related to SA response (Figure 3d–g, Supplementary Figure S4D and Supplementary Table S6). The broad involvement of SA-NEGs with various aspects of SA metabolism strongly suggest that this process influences heterosis.

Further, to determine whether SA-NEGs are regulated through DNA methylation, we performed bisulfite sequencing (BSseq) analyses of the different genotypes but failed to find any association of SA-NEGs with DNA methylation (Supplementary Information and Supplementary Figures S7 and S8).

### Endogenous SA levels determine plant size in WT and *ddm1* mutants

**Figure 4:**
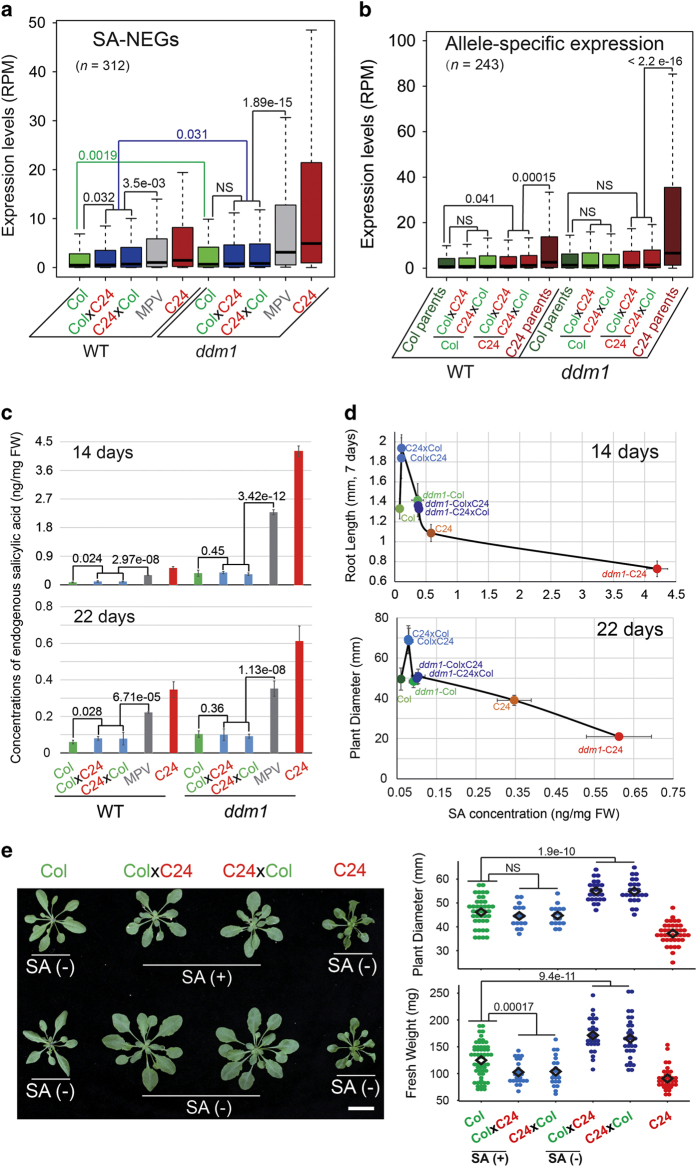
The expression of SA-NEGs correlates with endogenous SA levels. The expression pattern of 312 SA-NEGs were analyzed in different genotypes using boxplots. The differences between the corresponding pairwise comparisons were calculated using *t*-test. The green and blue lines indicated the differences of WT-Col/*ddm1*-Col and of WT-F_1_s/*ddm1*-F_1_s, respectively (**a**). Allele-specific gene expression was also analyzed for WT and *ddm1* using publicly available single-nucleotide polymorphism database between Col and C24 accessions (**b**). The endogenous SA concentration was measured in WT and *ddm1* genotypes (both parents and hybrid) at two developmental stages of 14 and 22 DAS (**c**). The dependence of root length and plant size on SA dose is shown (**b**, **d**). The effect of exogenous SA on heterosis performance of WT-F_1_ with foliar spray (see section Exogenous treatment of SA) was monitored and quantified in terms of plant size and FW (**e**).

To gain further insight into the regulation of SA-NEGs, we examined their expression levels by box plot analysis. As expected, the SA-NEGs were expressed at higher levels in the *ddm1* mutant compared with WT in parental and with hybrid backgrounds as well (Figures 3a,d–f and 4a). We noticed that the expression of SA-NEGs in WT-F_1_ was higher than in Col parents; however, no significant change in the expression of SA-NEGs was observed between *ddm1*-Col and *ddm1*-F_1_ (*P*>0.05), consistent with the comparable growth phenotypes of these plants (Figure 2a). To evaluate the expression of specific parental alleles in F_1_ hybrids, we selected 243 SA-NEGs that contain single-nucleotide polymorphisms, which distinguish the Col and C24 parent backgrounds. We evaluated the expression of these SA-NEGs by box plot analysis and found that the expression of the C24 allele, but not the Col allele, was significantly altered and reduced in the hybrids relative to the respective parent. In contrast to WT plants, SA-NEGs in *ddm1* mutants are expressed at similar levels from each allele in the Col parent and in the hybrid plants (Figure 4b). The *ddm1*-Col-like expression of SA-NEGs from both alleles in *ddm1*-F1 hybrid and Col parent plants may account for their similar growth phenotypes (Figure 2a).

As many SA metabolism-related genes were upregulated in *ddm1*-F_1_ compared with WT-F_1_, we hypothesized that different endogenous SA concentrations might underlie their distinct phenotypes. To test this hypothesis, we quantified SA levels in different genotypes. We found that the levels of SA (Figure 4c) correlated with the expression levels of SA-NEGs (Figure 4a) at both 14 and 22 days after germination. Maximum growth occurred at an optimal endogenous SA concentration observed only with WT-F_1_ hybrid plants (about 0.11 ng mg^−1^ fresh weight (FW)). Growth was reduced at sub-optimal endogenous SA concentrations (0.07 ng mg^−1^ FW, WT-Col) and supra-optimal concentrations (⩾0.3 ng mg^−1^ FW, WT-C24, all *ddm1* parent and hybrid plants) (Figure 4d). The SA level in *ddm1*-F_1_ increased by 3.55-fold compared with WT-F_1_ at 14 day-old seedlings, shifting it from optimal to supra-optimal and from BPH to MPH.

To further validate that loss of the BPH phenotype of *ddm1*-F_1_ arises from higher SA concentrations, we treated WT-F_1_ plants with exogenous SA and monitored growth performance. These treatments abrogated heterosis in WT-F_1_ hybrids, including heterosis in plant size and FW (Figure 4e). Moreover, low concentrations of SA improved the growth of Col plants but not C24 (Supplementary Figure S6), suggesting that SA is a hormetic regulator of plant growth. Together, these data suggest that heterosis in WT-F_1_ hybrids reflects an optimal level of SA metabolism, and that sub- and supra-optimal levels of SA negatively influence growth.

## Discussion

Col/C24 *Arabidopsis* hybrids are known to show heterotic phenotypes, including increased biomass and photosynthetic ability [29]. The extent of heterosis changes throughout the growth cycle of plant [30]. therefore, we selected early seedling stages and evaluated the contribution of different epigenetic regulators, such as some RdDM components and DDM1 (chromatin remodeler) in regulating biomass heterosis. The tested RdDM mutants maintained heterosis, at least during the early seedling stage. These results are consistent with recent work in *Arabidopsis* [14] and also with the observation in maize that loss of *MOP1* (modifier of paramutation1), an RDR2 ortholog in the RdDM pathway in maize, did not affect the hybrid vigor of B73 × Mo17 hybrids [15]. Unlike RdDM mutants, F_1_ hybrids generated in *ddm1* mutant backgrounds clearly showed reduced heterosis compared with WT-F_1_, with the BPH traits in WT-F_1_ changing to MPH in *ddm1*-F_1_.

NEGs, the subset of DEGs that deviate from the MPV, are routinely evaluated in hybrid progeny [1] and it has been proposed that NEGs may explain heterosis [31]. Indeed, NEGs have provided insight into the heterosis observed in Col/C24 hybrids towards abiotic and biotic stimuli [29]. We identified 312 functionally enriched NEGs related to SA (SA-NEGs) in WT/*ddm1* F1 hybrids. In general, a relatively higher expression of SA-NEGs correlated negatively with growth, except for WT-Col parental and WT-F_1_ hybrid plants (Figure 4). Indeed, WT-F_1_ plants display an optimal level of SA-NEGs expression associated with optimal growth; expression outside this range impairs growth.

We found that the SA-NEGs targeted by DDM1 are enriched for genes related to SA metabolism, including SA biosynthesis and its activation, SA downstream signaling and SA responsive genes. SA is a major phytohormone controlling the defence response, as well as growth and development in plants. Its biosynthesis occurs via two pathways: one from cinnamate, catalyzed by phenylalanine ammonia lyase, and the other from chorismate, catalyzed by isochorismate synthase (ICS) [32, 33]. ICS1 (also known as SID2) is the predominant form of ICS [34] and was an SA-NEGs gene. The SA-NEGs in the SA biosynthesis activation category included genes that function upstream of ICS1 or transcription factors that activate its expression. ALD1 is an aminotransferase mediating the biosynthesis of another immune regulatory metabolite, pipecolic acid, and FMO1 is an essential component of pipecolic acid downstream signaling. Although pipecolic acid positively regulates SA biosynthesis and signaling, there is a significant SA-independent component of ALD1/pipecolic acid/FMO1-mediated defense signaling [35, 36]. Mutations in these genes are known to attenuate SA accumulation in response to pathogen attack [33, 37, 38]. SA-NEGs were also enriched for genes that function downstream of SA, including SA-responsive genes. Interestingly, genes related to SA repression were absent, suggesting that SA-NEGs positively regulate SA signaling.

The enrichment for a wide range of SA-related genes in the set of SA-NEGs suggested that endogenous SA levels may differ between different genotypes and underlie heterosis. SA metabolism has been previously implicated in hybrid vigor in the contexts of the defence response and growth performance in *Arabidopsis* [8, 23]. Yang *et al.* [23] found that F1 hybrids with a higher level of SA than parents display heterosis for the defence response but not growth. Groszmann *et al.* [8] revealed that F1 hybrids with lower endogenous SA levels than MPV displayed heterosis for growth performance, which was compromised by SA treatment. Consistent with these findings, we observed a perfect overlap between SA-NEGs expression and SA concentration at two developmental stages. However, more interestingly, our data suggest hormetic modulation of hybrid vigor by SA. Increased, low endogenous SA concentrations stimulate growth (for example, WT-F_1_ compared with WT-Col) but, beyond a threshold, SA inhibits growth (for example, WT-C24 and all *ddm1* mutant plants). This type of SA dose-dependent growth phenotype with WT-F_1_ and Col supports the conclusion that SA is hormetic in promoting plant growth, consistent with previous supplementation [39–42] and genetic studies [43]. The endogenous SA concentrations of WT-Col and WT-C24 flank the optimal SA level for growth and the non-additive expression of SA metabolism genes in WT-F_1_ lead to optimal SA levels and the BPH phenotype. The allele-specific expression data revealed that *ddm1* mutants displayed increased expression of SA-NEGs from both alleles compared with WT, in particular from the C24 allele. Thus, both *ddm1* mutant parents display supra-optimal endogenous SA levels and SA-NEGs expression, which is maintained in the hybrid progeny. As a result, BPH is converted to MPH in *ddm1*-F_1_ due to the hormetic response to SA. Our findings that altered expression of SA metabolism and immune/defence response genes correlates with hybrid vigor suggest that the improved growth of hybrids may be recapitulated in parental plants by optimizing environmental stressors to induce endogenous hormetic responses. Hormesis appears to be induced by many endogenous biological processes, including stress, inflammation and cellular respiration, where a low level of potentially toxic byproducts ultimately improves performance [44–47]. We propose that some improved hybrid traits reflect the optimized, endogenous regulation of stress-induced hormesis.

We found that DDM1 is a major regulator of early stage growth heterosis in Col /C24 accession of *Arabidopsis*. In order to further dissect how *ddm1* drives BPH-to-MPH transformation in *ddm1*-F_1_, we performed transcriptome analysis and uncovered SA-NEGs, the differential NEGs enriched in the genes related to SA metabolism. Methylome analysis demonstrated that the SA-NEGs were mostly in the low- and unmethylated categories, which suggested that *ddm1* regulates the expression of coding genes by epigenetic mechanisms other than DNA methylation. DDM1-mediated regulation of coding genes has been previously demonstrated through generation of *ddm1*-induced epigenetic inbred lines, which show flowering time and root length phenotypes [48]. It has been shown that *ddm1* mutants display a genome-wide increase in transcription and in histone methylation marks associated with gene activation (H3K4me1/2/3) rather than repression (H3K9me2 and H3K27me3) [21, 49] implying that these histone modifications may be involved in regulation of SA-NEGs; however, other epigenetic marks might also be important, as reported previously [23, 50]. The role of epigenetic modifications in heterosis requires further investigation.

**Figure 5:**
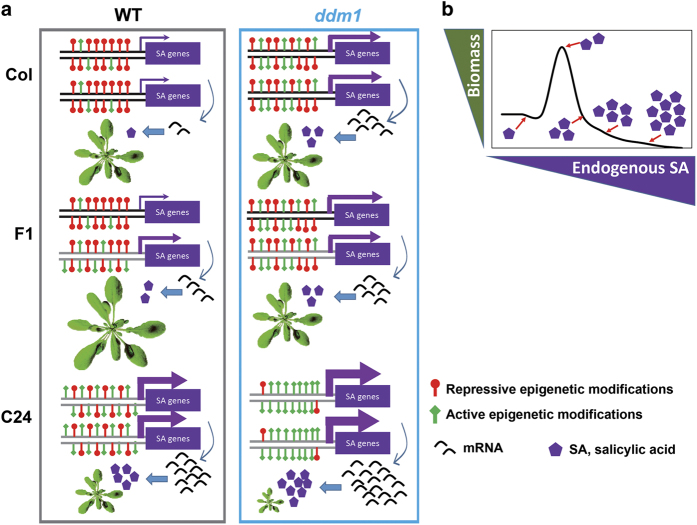
A working model for the proposed action of DDM1-mediated regulation of heterosis through SA metabolism. (**a**) The promoter status of SA-related genes, SA transcripts abundance, SA concentration and phenotype of parents and F_1_ in WT and *ddm1* mutant background. (**b**) The effect of dose-dependent SA concentration over plant growth. The F_1_ hybrids from WT Col and C24 possess SA gene expression/concentration in the optimum zone and, hence, BPH phenotype is seen. As *ddm1* increases the ratio of active (green bars) to repressive (red bars) epigenetic marks in promoter elements, SA-related gene expression and SA concentration is increased in *ddm1*-parents. Thus, F_1_ hybrids produced from the *ddm1* background display supra-optimal SA concentrations and hence MPH phenotype is seen.

In conclusion, the study revealed that the chromatin remodeler DDM1 is a major regulator of early seedling biomass heterosis in Col/C24 accessions of *Arabidopsis thaliana*. *ddm1* hybrids display elevated expression of NEGs that are functionally enriched for SA metabolism, leading to increased endogenous SA levels. We propose that SA is a hormetic regulator of plant growth, with increased growth associated with increased SA at low concentrations, but inhibition associated with high concentrations. WT-F_1_ hybrids generated from a cross of WT-Col and WT-C24 produce plants with optimal SA-NEGs expression, endogenous SA concentration and hybrid vigor. Our study links epigenetics to hormone metabolism and hybrid vigor through DDM1. Importantly, we propose that endogenous SA levels and SA-related gene expression can predict whether crosses between two genotypes will show growth heterosis (Figure 5).

## Materials and Methods

### Plant growth and phenotyping of different genotypes

The *Arabidopsis* seeds from WT Columbia-0 and C24 background were available in the lab. The epigenetic mutants in Col were procured from *Arabidopsis* Biological Resource Centre (http://www.biosci.ohio-state.edu/pcmb). The mutants in C24 background were from previous studies [24, 25]. Seeds were surface sterilized and then plated on Murashige and Skoog medium [51]. To ensure homogenous germination, seeds were kept at 4 °C. After 7 days of stratification, seeds were transferred to growth room and then allowed to grow in continuous, cool fluorescent white light (100 μE m^−2^ s^−1^) at 22 °C under long day conditions. The 7 day-old seedlings were transplanted on soil pots and at 25 day after sowed, phenotyping was performed in terms of plant width and leaf width. For root phenotyping, 12 day-old seedlings were transplanted on vertical plates and after 7 days increase in root length was measured.

### Generation of hybrids and genotyping

The F_1_ hybrids between Col-0 and C24 under WT and *ddm-1* background were generated as described previously [29]. The heterozygotic nature of produced hybrids was confirmed through genotyping according to particular mutant information. At least one natural variant site was selected between Col and C24, to confirm that F_1_ progenies were really from Col and C24 hybridization. The primers for genotyping and Chop-PCR are listed under Supplementary Table S2.

### RNAseq and data analysis

All the different genotypes were grown on plates in triplicates. At 14 day after sowed, entire seedling was collected, RNA was isolated and RNA sequencing (RNAseq) was performed on Illumina HiSeq 2000 platform (San Diego, CA, USA). The sequencing statistics of different RNAseq libraries can be found as Supplementary Table S4. The raw sequencing reads from all the runs were first trimmed for lo- quality regions and adapter sequences. Clean reads were aligned to TAIR10 *Arabidopsis* reference genome using TopHat2 software [52]. The resulted BAM alignment files were used as input to summarize the gene expression levels using HTSeq-count [53]. The low expressed genes were removed and only genes with an expression level of at least 1 read per million in at least three samples were retained for further analysis. The R package edgeR, which uses counts per gene from different samples as input and performs data normalization using trimmed mean of M-values method, was used to identify DEGs [54]. To identify NEGs, we compared the average expression value of two parents as MPV, with the expression values of F_1_ using the same algorithm and criteria as used for DEGs. The log2 transformed values of normalized gene expression (reads per million) was used to build an expression matrix. The matrix from different replicates from each genotypes was independently transposed (Supplementary Table S7), then prcomp function in R [55] was used to perform principal component analysis. The R package pheatmap (Kolde, 2011) was used to draw the heat map for gene expression of NEGs. The bedtools intersect was used to find the abundance of transposons and Fisher’s exact test was used to test for statistical significance. The single-nucleotide polymorphisms information was collected from publicly available database for Col and C24 (http://1001genomes.org/data/MPI/MPISchneeberger2011/releases/current/C24/Marker/C24.SNPs.TAIR9.txt) and allele-specific mapping was extracted from bam alignment file of F_1_ hybrids using asSeq [56], which was used as input for HTSeq-count to calculate the allelic expression level of NEGs.

The RNAseq data were validated using quantitative real-time PCR analysis, as described previously [57]. The primer details can be seen under Supplementary Table S3.

### GO analysis

To analysis the functional enrichments of NEGs, we performed the GO analysis using GOEAST (http://omicslab.genetics.ac.cn/GOEAST/). After we obtained the GO terms lists, the *P*-value was then adjusted by Benjamini and Hochberg method, to control the false discovery rate with<0.01 cutoff. The top 10 GO terms were showed in the figures as *P*-value bar plots.

### Measurement of SA contents in plants

The endogenous SA contents were measured using liquid chromatography–mass spectrometry. Approximately 25 mg fresh plant sample was homogenized with 500 μl acetone:50 mM citric acid extraction solvent (70:30, vol/vol). The content was centrifuged at 1 000 r.p.m. at 4 °C in dark for 3 h. The supernatant was separated and then evaporated using in a concentrator (Labconco) at −4 °C under vacuum for 1 h. The residual was added with 300 μl of diethyl ether and then centrifuged at 5 000 r.p.m. for 5 min. The upper organic phase was collected thrice. The pooled organic phase was dried at −4 °C under vacuum for 1 h and then dissolved in 200 μl of 50% acetonitrile. After centrifuging at 10 000 r.p.m. for 5 min, the supernatant was collected and used for liquid chromatography–mass spectrometry analysis. During extraction, SA-d4 was also added, which serve as internal standard.

### Exogenous treatment of SA

The exogenous supply to SA was given in the form of foliar spray or medium supplement. For foliar spray, SA was applied at two different developmental stages of the plant. Two different doses of SA such as 1 and 5 mM were used for plants at 15 and 22 day after sowed, respectively. The SA was prepared along with 0.01% Tween-20. The plants sprayed with Tween-20 alone served as control. For the medium supplement, *Arabidopsis* seedlings were grown on 1/2 Murashige and Skoog (MS) medium with different concentrations of SA. At 12 days after growth, seedlings were transplanted to soil for further phenotyping in the next development stages.

### BSseq analysis

All the genotypes were grown on plates for 14 days and then collected for BSseq analysis. The sequencing statistics of different BSseq libraries can be found as Supplementary Table S5. The raw reads were mapped to Col-0 TAIR10 *A. thaliana* genome with BSMAP aligner allowing up to four mismatches [58]. Only uniquely mapped reads were used for subsequent calculation. Only cytosines with a depth of at least four were kept for the determination of cytosine methylation levels [59]. We used the DNA methylation levels of gene body, upstream (2 kb) and downstream for each annotated, to build the DNA methylation matrix (Supplementary Table S8), then used the prcomp function in R [55] for principal component analysis.

### Statistical analysis and sequencing data deposition

The experiments during the entire course of the study were carried out in a completely randomized design. All the experiments were repeated at least twice to check reproducibility. Student’s *t*-test was performed to determine the significant difference between treatments using R statistical computing package [55]. Genome-wide DNA methylation profile was visualized in Integrative Genomics Viewer [60] or Integrated Genome Browser [61].
