# Supplementary material for: The chromatin remodeler DDM1 promotes hybrid vigor by regulating salicylic acid metabolism
Source: Cell Discov. 2016 Aug 9;2:16027–. doi: 10.1038/celldisc.2016.27 (PMC4977722; doi:10.1038/celldisc.2016.27)
Supplement: Supplementary information [file celldisc201627-s1.pdf]

Supplemental Table S1. Genetic information for mutant alleles of the RdDM pathway in Col and C24 background, Related to Figure 1.

Gene Name	Gene ID	Col Alleles	C24 Alleles
RDR2	AT4G11130	SALK_059661	CDS G405A/W135*
DMS3	AT3G49250	SALK_068723C	T-DNA insertion at 21bp(from ATG)
DRD1	AT2G16390	CDS_G2268A/W756*	CDS_2286 35bp deletion
RDM1	AT3G22680	CDS_C307T/R103*	CDS_T456A/Y152*
NRPD1	AT1G63020	SALK_128428	T-DNA insertion in the 8th exon
NRPE1	AT2G40030	SALK_029919C	gDNA_5189(from ATG), 31bp deletion
AGO4	AT2G27040	SALK_071772	gDNA_4641(from ATG), 6 bp deletion
AGO6	AT2G32940	SALK_031553	T-DNA insertion at 3104 bp(from ATG)
KTF1/RDM3	AT5G04290	SALK_001254	T-DNA insertion in the 1st intron

Supplemental Table S2. Primers for genotyping and Chop-PCR, Related to Figure 1 and 2.

Gene Name	Gene ID	Alleles	Forward (5' > 3')	Reverse (5' > 3')	Enzymes	Applied Alleles	Products
NRPD1	AT1G63020	Col	GATCTGTTCAGCTTGCTCGTC	TTAATGTTCTTCATGCGGGAC	N/A	N/A	N/A
NRPE1	AT2G40030	Col	ATTTCTTCTTTGATGGGGGAG	TGTCGTGGATATGACCATTTG	N/A	N/A	N/A
DRD1	AT2G16390	Col	TCTGAGCAGCGTGAGTGGTCA	GGGTCGAAACGCTCGCCCAA	N/A	N/A	N/A
RDM1	AT3G22680	Col	TCAGGAAAGATTGGGTCAATG	GAAACCTCCGTTGGAAGATTC	N/A	N/A	N/A
KTF1/RDM3	AT5G04290	Col	GTGGGAAAGGAGAAGGATCAG	AAACGCATGAAAACAAACCTG	N/A	N/A	N/A
AGO4	AT2G27040	Col	TTCTCCAGCTGGCTAGCTATG	CCCAGAAAGGTGACATCTTTG	N/A	N/A	N/A
RDR2	AT4G11130	Col	CTGATCGCGAGATTTCAGTTC	AGAAGATTGGAGCAAGCTTCC	N/A	N/A	N/A
AGO6	AT2G32940	Col	GTCTGGGAAACCCAAAGAGAC	ACCGGAAGAACTACCACCATC	N/A	N/A	N/A
DMS3	AT3G49250	Col	GAAACTCAGAATGGAGGAGGC	TGATGATTCCTCCATCCAAAG	N/A	N/A	N/A
NRPD1	AT1G63020	C24	TGCTGTTTGCCGTTCCGTGGT	AGCGCGCTGTAAGCTGCCTC	N/A	N/A	N/A
NRPE1	AT2G40030	C24	TCCTACAATGCCACCGATCCCGA	ACTGCTCCCCCATCAAAGAAGAAATCG	Deletion	Mutant	110/75
DRD1	AT2G16390	C24	TCTGAGCAGCGTGAGTGGTCA	TGCGAGATGCTCCAACAAGCG	Deletion	Mutant	115/80
RDM1	AT3G22680	C24	CCCGACGAATCGTGGCTCGTT	GCAGCCACAGCGAGAAACTTGC	MaeIII	Mutant	350+276
KTF1/RDM3	AT5G04290	C24	GCCCTTTTCTCCCTTGCCAGCC	ACGGAGGCAGCTCCGACGAA	N/A	N/A	N/A
AGO4	AT2G27040	C24	GGTTCTGAATATTGAACTTGATCAGATCATCGAGC	TGCAAGCCTGAGGAAAGAGAAATGC	AluI	Wild Type	80+ 35
RDR2	AT4G11130	C24	ACGACATCGTTTTGACTGTTGGGT	TGCATCAATCTCAGAAGCGTCACCA	ApoI	Mutant	140+125
AGO6	AT2G32940	C24	TCCACACACAATGCATCTGCCCA	GGGCCAGCATTTTGAACCAACAACC	N/A	N/A	N/A
DMS3	AT3G49250	C24	AGCGTGAGCAATGCCTCCTCC	AGAAAACCAGTACGGCGTTCGACAA	N/A	N/A	N/A
DDM1	AT5G66750	*ddm1-1* (Col)	TCGATGGCAGTGTGAAGCTGGA	TCGTCTGCCCGATTCTGTGGC	NlaIII	Wild Type	242+159
DDM1	AT5G66750	*ddm1-9* (C24)	TCGTGACAGATCTCTACCCTCCTGT	CTACGAGCCATGGGTTTGTGAAACAAA	ApoI	Mutant	100+32
DDM1	AT5G66750	*ddm1-10* (Col)	CCTAAACCGGGTTTGCTTTAC	ATTGTTGCCTCATGAGATTGG	N/A	N/A	N/A
DDM1	AT5G66750	*ddm1-14* (C24)	AGGGCCACAGGTTGAAAAACCAC	TGTCAAAAAGCTGCCACCAGTGT	MboI	Mutant	147+118

DDM1	AT5G66750	*ddm1-15* (C24)	TGCCACAGAATCGGGCAGACG	AGGCAACAATCTCAATGGTTTGAAGT	MnlI	Wild Type	63+63
DDM1	AT5G66750	*ddm1-16* (Col)	GATGACCAGGTCCTTATCTTCTCCCCATG	TCCAGCTTCACACTGCCATCG	NcoI	Wild Type	83+30
Chop-PCR	5S rDNA	N/A	AATGGCAGCCCACGCAAGCA	ATGCCCACGGAGCCCCAACT	N/A	N/A	N/A
T-DNA	LBb1.3	N/A	ATTTTGCCGATTTCGGAAC	N/A	N/A	N/A	N/A
T-DNA	C24_LB	N/A	TTGACCATCATACTCATTGCTG_	N/A	N/A	N/A	N/A

Supplemental Table S3. Primers for qPCR validation, Related to Figure 3.

Gene_ID	Forward (5' > 3')	Reverse (5' > 3')
AT1G15890	GTACGAAGCTGCCTTAAGCTACCG	TTCATTGGCATGTCGCGGAAAC
AT1G18860	TGTGAGACACCAACGATGAACGAC	AAGAAGCTGCAATGGTGCAACG
AT1G58807	CATGCCACTTCTTCGTACTCTCG	CCTAGAGTCGGCAATGGATCTTTC
AT1G61190	ACGTATGGATCCTCCAGAACAGG	TTTAGACGACGTACTAGCGGTTTG
AT1G61300	ACTGGAGTCCTCTCCACTTTCCAC	TCCCAGTCCTGGAGGATACATACG
AT1G61310	GCAGAGGGAAATGGAAGATCTGAG	TTGATGGCGTGACTCCTCTCTC
AT1G62630	TTCCAGAAGTGGCAAGCAAGGC	TCGTCTGCCCATTTCACACCTTTC
AT1G64280	AACGATTCTTCCCGCGCTGTTC	TTCTCCGCAAGCCAGTTGAGTC
AT1G75040	CCAAGTGGGACATGGTCAG	ACTTGTGTGCTGGGAAGACA
AT2G14610	AGCTTCCTTCTTCAACCACACAGC	TGGCAAGGTATCGCCTAGCATC
AT2G21900	AACGGGTAGTCCATTTCCAAGGC	ACAGAAGGGCTTGGGTGGTTATG
AT2G40750	CACTGCTCAGAACCATGTCAATGC	TGCTGTCACACTGCTGGTGTTG
AT2G46400	ACCTGCTGCTGTTGAGAATTCCG	ACGACCACAACCAATCCTGTCC
AT3G01345	TTGCTGCCCACACCAAGTATCG	ACCAGCCCAAACAGAGGTAGAG
AT3G04720	ATCACCCACAGCACAGAGACAC	AGCAATGCCGCTTGTGATGAAC
AT3G14460	ATCGAGACGATTCCTGGTGGAG	GGTGTAAGCTTGTCGCAAAGGC
AT3G22600	AGTCGCTGTAATACCGGTGGTG	CCTGGTCCTGAAGAGTTTGGTG
AT3G44400	ATTCTTCGCCGGATCTCTCGTC	TCGAATCCATCGAATGACTCAAGG
AT3G46730	AGGCCATGGATTAGATGAGTTGCG	TAATCGGCCCTTCACAGTCAGC
AT3G56400	TGAGCTCGAACCCAAGATGTTCAG	TGCTCTTGGGAGTTTCTGCGTTG
AT3G57260	GCCATCTCATTGTTGACTACCAATTT	ATCAATGGCCGAAACAAGCA
AT4G09420	AAGAACTCGCAGCGTCCTTCAG	CGTAGTCTCGTCGTCATGGATTCG

AT4G12520	TGCACCGCCCTAAAGGCTAAAG	TCAAGCACATACGAAGCCGGAAG
AT4G16860	CCCTGCTGCATTGAGGATTTAACG	AAGTCGGCGACCATTAGACTTG
AT4G16960	AAGATGCGATGCGGCGTACAAC	TCCAGATTTGGAGCGGTCAATCC
AT4G16990	TGTGTGTTCGCCTAATGGTAAGGG	TGCAGGCATATGTGCTTGAGG
AT4G19500	TCAGGAAATGCCGAGTCTTCCG	CAGGGATTGAGAGGTGATTTCTGC
AT4G19510	ACCACGGAAACTTGGATCTGACC	TCTTCGCTCCATTTGAAGACTGG
AT4G22470	TGGTCGTAGGATTCCACAAGGC	GTGGTGGTGGTGAGATTGTTGG
AT4G23810	ATCCCGGCAGTGTTCCAGAATC	AGAACCTCCTCCATCGGCAAAC
AT5G01080	ATCCAACAGCCTCCTCTTTGGAG	AGACGGCTCGAGCAAACAATG
AT5G01900	CAACCAGCTGCTCATCATGGAC	ACTTGGCCAAATCCTCCCTTTCC
AT5G05400	AGAAGGGTCAAGCAGCAACGAG	AAGGATATGTCCCTCCGAACCC
AT5G11250	TCTATCCATGAGCCTGGACAACG	ACTACCTGCTGCATCACCATTC
AT5G13080	AGTGGACCAAGAAGTGGTCGTG	TTCTCGATGGGATGCGAATGCAC
AT5G24110	TCTCGGAGCCAAATTTCCAAGAGG	TCCTCGGTAACTGATCTCAAGGAG
AT5G24480	CAAGCCATGTGGCAAAGGACAC	AGGGAGCTTTGGTGTAGTCACG
AT5G24770	TTGGCAATATCGGAGATCAAT	GGGACAATGCGATGAAGATAG
AT5G26170	AGAACAGCCCACATCCAAGAAAC	TCTTCACGGGACAGCCATCAAC
AT5G35760	ACGCCATTATGATCCCATCTCCAC	GGTAATTGGCGGGTAAGGAGGTAG
AT5G35890	TTGACAACCCGTGCTCTCACTG	CCATGAAACTTGAGGGCCAATGC
AT5G38350	AGAACTTGTGGCAAGGAAATCAGC	AGCTCCACCAAACTTATGCATCC
AT5G44420	GTGCCAAAGTGAGGTGTAACAA	CGTGTGTATGCATGATCACATC
AT5G45490	ATGGACAAACCGACTCATCGAACC	TCTTGAGCCAAGAGGCTGAGTC
AT5G46350	CACGACTCAGAAGTGCAACGTG	AGGTTGTGATGACGACCGTTGG
AT5G46900	CCCAAGAGCCACCACAAGAAAC	AGCGTCTTTACAAGTGGGTTTAGG
AT5G47260	GTGGAGAAACTGCCACTGCAAAC	TTGCGTGTGTTCCCGATTGGAC
AT5G64810	GACGGGTCATCGAGTTGCATTTAG	ACCGAGCAACCTTCACTTGAGC

Supplemental Table S4. RNAseq data set, Related to Figure 3.

Sample Name	Raw_Reads	Clean_Reads	Data_size(bp)	Depth(X)	%	Unique Mapped_Reads	%
Col_rep1*	-	52,288,194	4,705,937,460	92.27	-	48,227,445	92.23%
Col_rep2	44,724,802	42,287,710	4,271,058,710	83.75	94.55%	40,320,043	95.35%
Col_rep3	42,998,300	42,117,826	4,253,900,426	83.41	97.95%	40,286,420	95.65%
ColxC24_rep1*	-	53,605,030	4,824,452,700	94.60	-	47,787,869	89.15%
ColxC24_rep2	45,918,634	43,998,964	4,443,895,364	87.14	95.82%	40,279,610	91.55%
ColxC24_rep3	43,703,976	42,865,730	4,329,438,730	84.89	98.08%	39,847,384	92.96%
C24xCol_rep1*	-	51,201,594	4,608,143,460	90.36	-	44,183,216	86.29%
C24xCol_rep2	47,882,216	45,894,536	4,635,348,136	90.89	95.85%	43,046,511	93.79%
C24xCol_rep3	48,116,248	47,346,672	4,782,013,872	93.76	98.40%	43,910,050	92.74%
C24_rep1*	-	51,313,670	4,618,230,300	90.55	-	43,376,625	84.53%
C24_rep2	50,471,752	48,508,544	4,899,362,944	96.07	96.11%	44,286,584	91.30%
C24_rep3	45,103,910	44,254,552	4,469,709,752	87.64	98.12%	39,826,590	89.99%
ddm1-Col_rep1*	-	54,882,840	4,939,455,600	96.85	-	48,491,886	88.36%
ddm1-Col_rep2	46,602,710	45,112,596	4,556,372,196	89.34	96.80%	42,922,242	95.14%
ddm1-Col_rep3	44,302,852	43,579,340	4,401,513,340	86.30	98.37%	41,282,793	94.73%
ddm1-ColxC24_rep1*	-	55,123,730	4,961,135,700	97.28	-	47,551,890	86.26%
ddm1-ColxC24_rep2	49,595,054	48,251,126	4,873,363,726	95.56	97.29%	44,378,447	91.97%
ddm1-ColxC24_rep3	41,458,168	40,721,794	4,112,901,194	80.65	98.22%	37,118,404	91.15%
ddm1-C24xCol_rep1*	-	51,949,000	4,675,410,000	91.67	-	46,078,553	88.70%
ddm1-C24xCol_rep2	44,034,532	42,572,010	4,299,773,010	84.31	96.68%	38,997,801	91.60%

ddm1-C24xCol_rep3	37,013,554	35,564,288	3,591,993,088	70.43	96.08%	32,416,286	91.15%
ddm1-C24_rep1*	-	54,239,524	4,881,557,160	95.72	-	45,410,388	83.72%
ddm1-C24_rep2	45,164,888	43,653,310	4,408,984,310	86.45	96.65%	38,205,986	87.52%
ddm1-C24_rep3	42,170,418	40,024,474	4,042,471,874	79.26	94.91%	34,557,911	86.34%

Supplemental Table S5. BSseq data set, Related to Figure 3 and 5.

Samples	Raw reads	Mapped Reads	Mapped rates	Depth	Conversion Rates *	C	CG	CHG	CHH
Col	49,025,828	45,083,334	91.96%	34.11	99.60%	6.75%	26.74%	8.56%	2.38%
ColXC24	51,362,438	43,716,788	85.11%	33.07	99.63%	8.91%	28.56%	10.91%	3.52%
C24XCol	44,700,556	38,811,690	86.83%	29.36	99.66%	9.60%	30.75%	11.79%	3.71%
C24	48,760,236	39,186,398	80.37%	29.65	99.61%	7.05%	24.37%	8.46%	2.68%
ddm1-Col	46,370,212	41,679,868	89.89%	31.53	99.67%	3.44%	12.17%	3.53%	1.69%
*ddm1*Colx24	52,098,186	44,923,246	86.23%	33.99	99.65%	3.59%	10.28%	3.14%	1.97%
ddm1-C24xCol	52,116,108	44,850,052	86.06%	33.93	99.64%	3.18%	10.12%	2.88%	1.63%
ddm1-C24	46,234,356	36,653,222	79.28%	27.73	99.61%	2.55%	8.34%	2.55%	1.22%

Supplementary Text

DEGs explained differential phenotypes between parents but could not justify heterosis

To understand the molecular mechanisms that underlie heterosis, we performed whole genome transcriptome (RNAseq) and DNA methylome (bisulfite sequencing, BSseq) analysis of 14 d old seedlings of different genotypes. The results were first analyzed using Principal Component Analysis (PCA). Three biological replicates for each genotype clustered together in RNAseq (Supplemental Figure S4A, Table S7), suggesting that the library preparation and sequencing were highly reproducible. We identified differentially expressed genes (DEGs) between Col and C24 parents by RNAseq, and confirmed 48 randomly selected DEGs by qRT-PCR. We observed a very strong correlation between qRT-PCR and RNA-Seq (Supplemental Figure S4C, Pearson correlation coefficient = 0.85, p = 5.18e-09), which validated RNAseq results. The PCA plot indicated that the expression patterns in WT-F_1 and *ddm1*-F1 hybrids were distinct from their respective parents (Supplemental Figure S4A). The PCA plot of BSseq data revealed a similar pattern with that of RNAseq for the WT series. In contrast, the *ddm1*, F_1 hybrids overlapped with the *ddm1*-Col parent, whereas the *ddm1*-C24 parent represented a separate group (Supplemental Figure S4B, Table S8).

To investigate the molecular basis of BPH-to-MPH conversion in *ddm1*-F_1, we identified differentially expressed genes (DEGs) in different genotypes. A total of 12

groups of pair-wise comparisons were made in F_1 and parents in WT and *ddm1* mutant (Supplemental Figure S5A). The total number of DEGs between F_1 and parents was much greater in the *ddm1* backgrounds (4411) than in the WT backgrounds (1684), consistent with a hypermethylation function for DDM1. *Ddm1* mutants become hypomethylated, which in turn activates gene transcription, leading to higher number of DEGs. Further, a majority of WT DEGs (73.9%, 1246/1684) were shared with *ddm1* DEGs, implying that these additional DEGs are responsible for *ddm1* effect on plant phenotype. In WT, a large number of DEGs (1666) were observed in Col Vs. C24 which explained the growth difference between Col and C24. From WT-F_1 vs. Parent (ColxC24 Vs. Col and ColxC24 Vs. C24) comparisons, we identified a total of 574 DEGs; a large proportion of which was shared with Col/C24 DEGs (561/574). Only a small proportion of these DEGs (8/574) were differential in both Col and C24 (Supplemental Figure S5B). This was not consistent with the observed phenotypes, as F_1 showed biomass heterosis compared to both the parents. The *ddm1*-Col vs. *ddm1*-C24 showed 4115 DEGs which were much higher than that of WT, indicating that *ddm1* mutation could enlarge the variance between parents. This was also supported from the PCA plot (Supplemental Figure S4A) and phenotypic observations (Figure 2B). Similar to WT, even *ddm1* mutant data revealed that a majority F_1/parents DEGs was shared with Col/C24 DEGs and also only few genes in F_1 are differentially expression as compared to both the parents (Supplemental Figure S5C). Thus, although DEG analysis provided insight into the molecular basis for differential phenotypes between parents; it could not explain the

occurrence of heterosis in WT or why the *ddm1* mutation shifts BPH to MPH.

Expression of SA-NEGs is not mainly regulated by DNA methylation

We classified the 312 SA-NEGs into three groups based on the DNA methylation levels in putative promoter regions (2 kb up-stream regions of transcription start site (TSS)): high-methylation (n=43, mC > 0.1), low-methylation (n=99, 0.1>mC > 0.01) and unmethylated (n=170, mC < 0.01) (Supplemental Figure S7 and Table S6), and subsequently determined transposon abundance in their promoter regions, given that DDM1 typically targets these regions (Supplemental Figure S8A). RNAseq and BSseq data for each group were compared independently, confirming the inverse co-relation between promoter methylation and gene expression. The high-methylation group showed significantly lower spearman co-efficiency -0.47 (p < 2.2e-16), indicating that methylation represses gene expression. The promoters from the high-methylation group were enriched in transposons (Supplemental Figure S8A). The low-methylation group exhibited a lower negative association of expression with DNA methylation of promoters (rho = -0.24; p = 2.8e-16) than high-methylation group, and no significant correlation between methylation and gene expression was seen for the unmethylated group (P> 0.05). For a few representative genes, promoter methylation and gene expression profiles were visualised using the IGV server independently for high- (Supplemental Figure S8B) and low- (Supplemental Figure S8C) methylated group. The SA-NEGs related to the SA pathway were enriched in the low-methylation and unmethylated category (Figure

3D-F, S4D).

Supplementary Figures Legends

Supplemental Figure S1. Genotyping and Phenotyping of RdDM mutants

(**A**) Genotyping of *rdr2*. The polymorphic sequence was amplified and digested with appropriate restriction enzyme (Supplement file 1, 5, 6 and 8). The products were ran on 4% agarose gel. (**B**) Genotyping of *dms3*. Amplification was carried out with specific primers located or outside T-DNA inserts, and amplicons were run on 1.5% agarose gel. Genotyping of *drd1* (C), *rdm1* (D) *nrpe1* (F) and *ago4* (G) was performed as described for *rdr2*. Genotyping of *nrpd1* (E), *ago6* (H) and *rdm3* (I) was performed as per *dms3*. The LP, RP denote left, right genomic primers; BP stands for T-DNA border primer. Variation means a single nucleotide polymorphism (SNP) or an indel between Col and C24, which was used to examine whether hybridization between Col and C24 were successful. (J) The phenotyping of F1 and reciprocal F1 produced under different RdDM mutant background was performed and data was quantified in the terms of leaf width (n > 6).

Supplemental Figure S2. Genotyping of *ddm1* mutant alleles

Panel A-G represents genotyping of different *ddm1* alleles. Sequence polymorphisms and T-DNA inserts were used to identify *ddm1* alleles in Col, C24 and their F1 hybrids (Supplement files 7 and 8). In (A, B), more than two variations were applied to identify F_1 hybrids, and the chromosome on which a variation is located is shown on the right. The panel H represent the chop-PCR result of 5s rDNA for different ddm1 alleles. The 5S rDNA region was amplified from DNA templates digested by HpaII, and products were run on a 2% agarose gel. The de-methylation of 5S rDNA is considered as an important indicator of functional *ddm1* alleles. The loss of 5S rDNA methylation in all the three newly generated *ddm1* allele (*ddm1-14*, *ddm1-15* and *ddm1-16*) confirms their functional nature.

Supplemental Figure S3. The early seedling growth heterosis was impaired in F1 hybrids resulted from crosses between different *ddm1* mutated alleles in Col and C24 backgrounds

(**A**) The different *ddm1* mutated alleles in Col (green) and C24 (red) backgrounds. (B) F1 hybrids showed reduced heterosis in 18-22 DAS in *ddm1* background. (**C**) The heterosis was maintained in 18-22 DAS when different *ddm1* mutated alleles were crossed with WT alleles. (**D**) Phenotypic representation of impaired heterosis in *ddm1* background at 18-22 DAS. (Scale bar = 1 cm) (**E**) Heterosis was presented in F1 hybrids from crosses between *ddm1* mutant and WT. F1, F1 offspring from ColXC24 crosses; rF1, F1 offspring from reciprocal C24XCol crosses. (**F**) Similar growth pattern displayed between F1 offspring from cross between two WT parents and heterozygous F1 hybrids from cross between *ddm1* and WT parents.

Supplemental Figure S4. Principal component analysis (PCA) of transcriptome and DNA methylation data, real-time PCR based validation of RNAseq data and SA related genes

The PCA analysis was performed on the RNAseq (A) and BSseq (B) data. Different genotypes under WT and *ddm1* are represented as solid dots and circles, respectively. The expression of 48 randomly selected DEGs was performed through real-time qPCR and data was correlated with that of RNAseq (C). The SA-NEGs related to SA metabolism were classified on the basis of their involvement with SA responsive genes (D). The letter 'O' and 'D' in brackets before gene IDs indicated that the genes were from 'overlap' or '*ddm1* specifc' groups in C respectively. The letter 'H', 'L' and 'U' in brackets after gene names indicated that the gene were from 'High-methylation', 'Low-methylation' or 'Unmethylated' groups in Figure 6.

Supplemental Figure S5: Pair wise analysis of differentially expressed genes (DEGs) in different genotypes of WT and *ddm1* mutant background.

(A) Barplots showing the number of DEGs related to four genotypes of parents and F1 hbyrids in WT and *ddm1*. **(B)** Venn charts showing the overlapping DEGs F1/parents and Col/C24 in WT. **(C)** Venn charts the overlapping DEGs of F1/parents and Col/C24 in *ddm1*.

Supplemental Figure S6. Hormetic effects of exogenous SA on seedling growth. Arabidopsis seedlings from Col and C24 were grown on 1/2 MS medium with different concentrations of salicylic acid, as indicated (mol/L). At 12 d after growth seedlings were transplanted to soil. At 6 d (Col) or 8 day (C24) after growth, their growth pattern was documented and quantified in terms of fresh weight and plant size (n = 40-45 seedlings, scale bar = 1 cm).

Supplemental Figure S7. Analysis on the relationship between DNA methylation of promoters and expressions of SA-NEGs. The SA-NEGs were classified into three groups on the basis of methylation levels in putative promoter elements (up-stream -2kb from transcript start site (TSS)) in the Col parent: high-methylation, mC > 0.1; low-methylation, 0.1> mC > 0.01; unmethylated, mC > 0.01. The normalized gene expression levels for each group is also shown (A)

Supplemental Figure S8. Transposons enrichment profiles and examples of different sub-groups of SA-NEGs. Related to Figure 6.

(A) Transposons enrichment profiles of SA-NEGs. **NEGs**, sum of the corresponding SA-NEGs; **Random**, the same number of genes randomly selected in the genome, 1000 times calculation. (B and C) Promoter DNA methylation and gene expression profiling of representative High (B) and un-methylated (C) NEGs in different genotypes in WT and *ddm1* background.

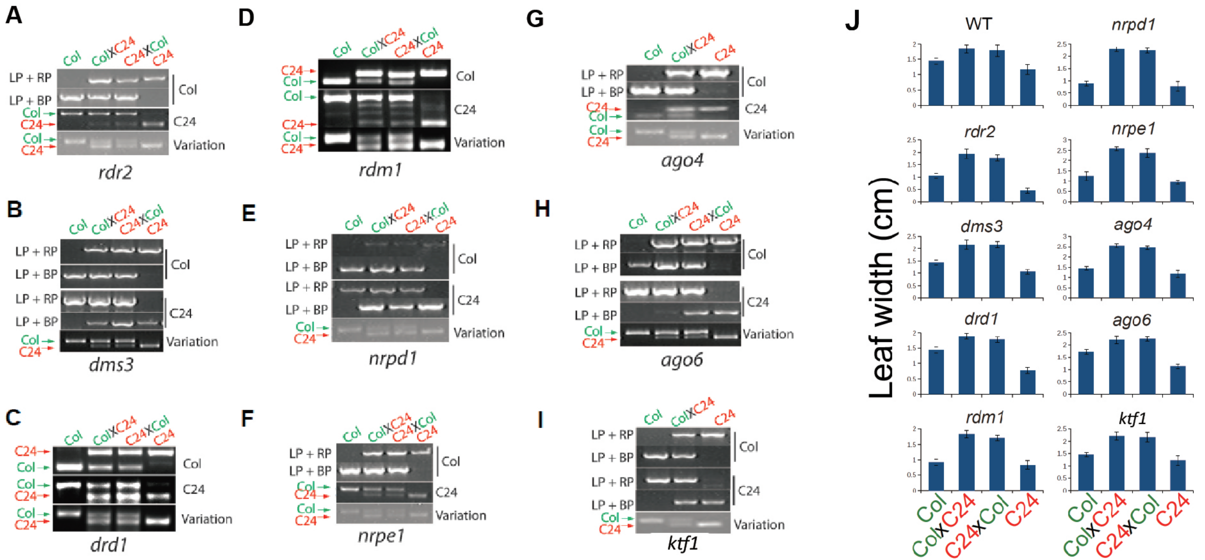
A
Col
ColXC24
C24XCol
C24
LP + RP
LP + BP
Col
C24
Variation
rdr2
B
dms3
C
drd1
D
rdm1
E
nrpd1
F
nrpe1
G
ago4
H
ago6
I
ktf1
J
WT
nrpd1
rdr2
nrpe1
dms3
ago4
drd1
ago6
rdm1
ktf1
Leaf width (cm)

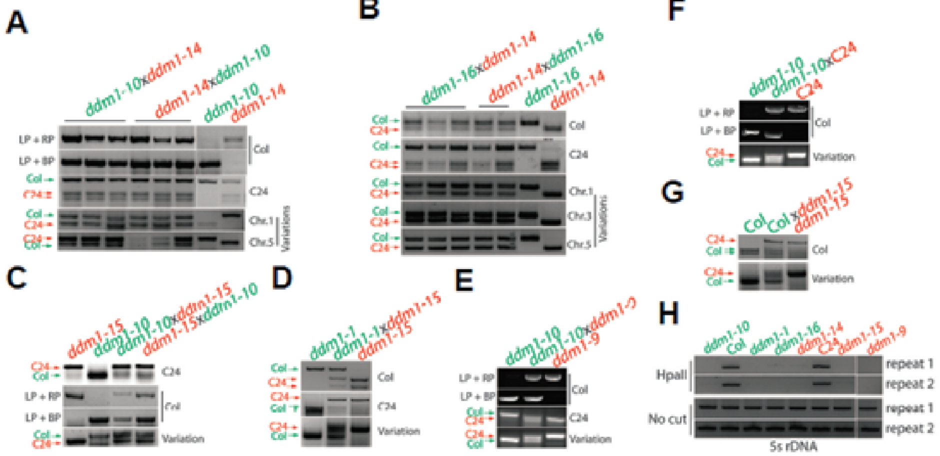
A
ddm1-10xddm1-14
ddm1-14xddm1-10
ddm1-10
ddm1-14
LP + RP
LP + BP
Col
C24
Chr.1
Chr.5
Variations
B
ddm1-16xddm1-14
ddm1-14xddm1-16
ddm1-16
ddm1-14
Chr.1
Chr.3
Chr.5
Variations
C
ddm1-15
ddm1-10
ddm1-10xddm1-15
ddm1-15xddm1-10
Variation
D
ddm1-1
ddm1-1xddm1-15
ddm1-15
E
ddm1-10
ddm1-10xddm1-9
ddm1-9
F
ddm1-10
ddm1-10xC24
C24
G
Col
Colxddm1-15
ddm1-15
H
ddm1-10
Col
ddm1-1
ddm1-16
ddm1-14
C24
ddm1-15
ddm1-9
HpaII
No cut
repeat 1
repeat 2
5s rDNA

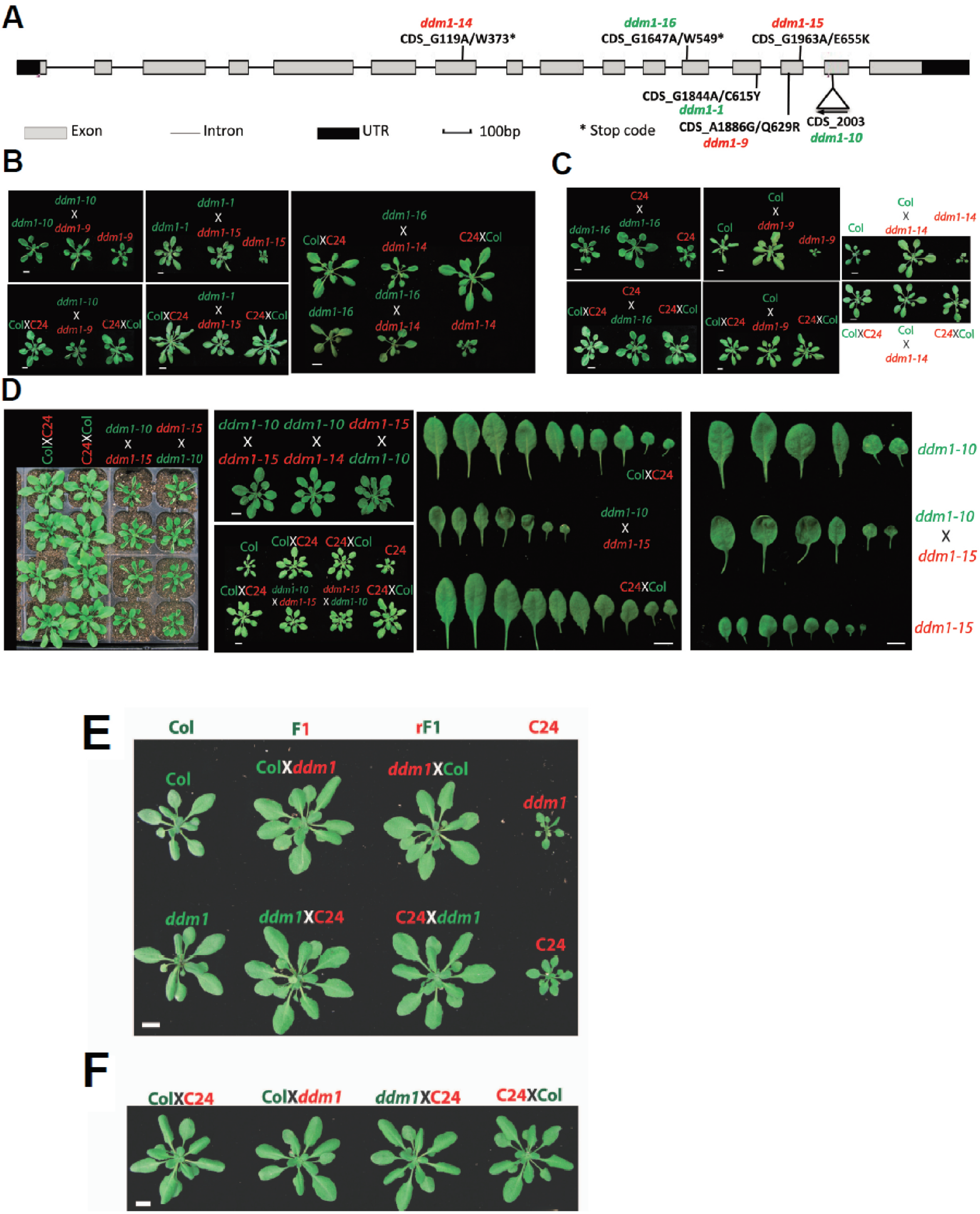
A
ddm1-14
CDS_G119A/W373*
ddm1-16
CDS_G1647A/W549*
ddm1-15
CDS_G1963A/E655K
CDS_G1844A/C615Y
ddm1-1
CDS_A1886G/Q629R
ddm1-9
CDS_2003
ddm1-10
Exon
Intron
UTR
100bp
* Stop code
B
C
D
E
Col
F1
rF1
C24
ColXddm1
ddm1XCol
ddm1
ddm1XC24
C24Xddm1
F
ColXC24
ColXddm1
ddm1XC24
C24XCol

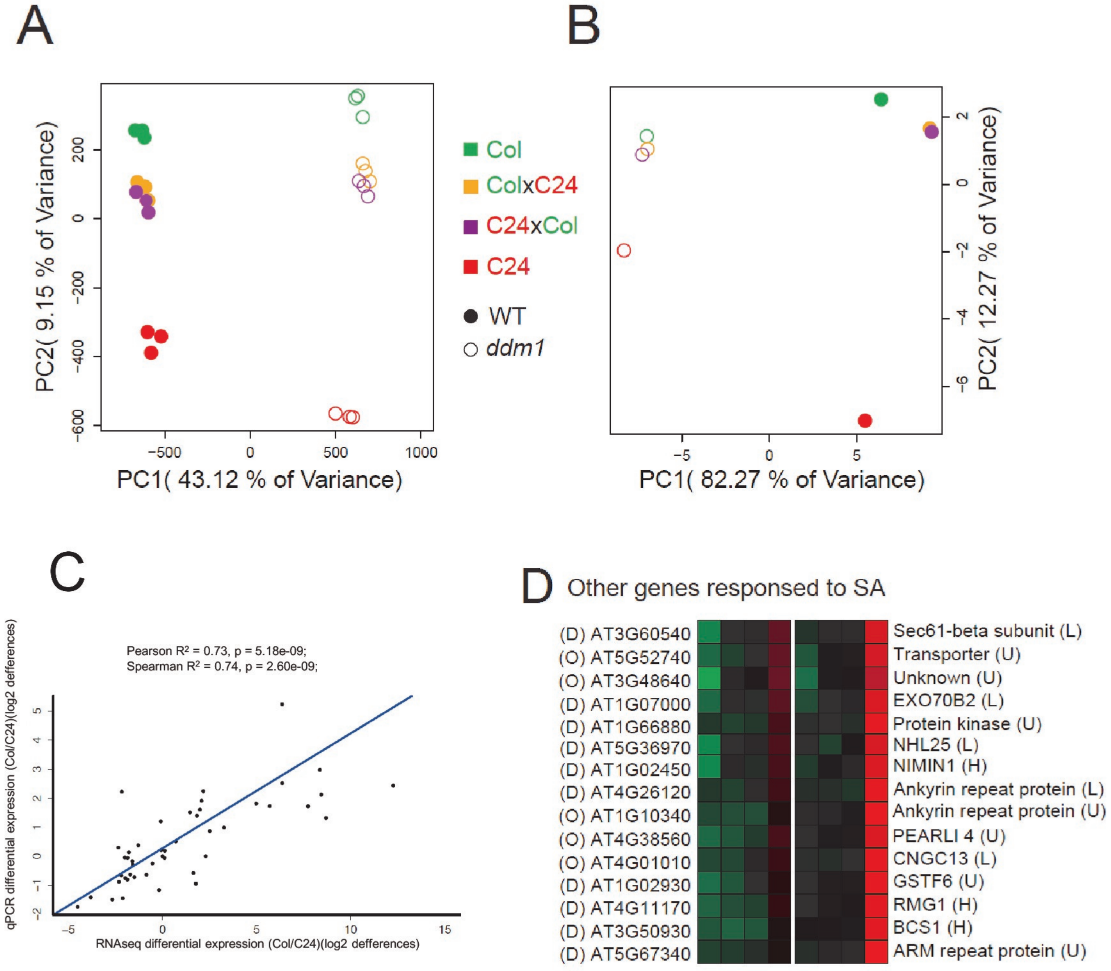
A
B
PC2(9.15 % of Variance)
PC1(43.12 % of Variance)
Col
ColxC24
C24xCol
C24
WT
ddm1
PC2(12.27 % of Variance)
PC1(82.27 % of Variance)
C
Pearson R2 = 0.73, p = 5.18e-09;
Spearman R2 = 0.74, p = 2.60e-09;
qPCR differential expression (Col/C24)(log2 defferences)
RNAseq differential expression (Col/C24)(log2 defferences)
D Other genes responsed to SA
(D) AT3G60540 Sec61-beta subunit (L)
(O) AT5G52740 Transporter (U)
(O) AT3G48640 Unknown (U)
(D) AT1G07000 EXO70B2 (L)
(D) AT1G66880 Protein kinase (U)
(D) AT5G36970 NHL25 (L)
(D) AT1G02450 NIMIN1 (H)
(D) AT4G26120 Ankyrin repeat protein (L)
(O) AT1G10340 Ankyrin repeat protein (U)
(O) AT4G38560 PEARLI 4 (U)
(O) AT4G01010 CNGC13 (L)
(D) AT1G02930 GSTF6 (U)
(D) AT4G11170 RMG1 (H)
(D) AT3G50930 BCS1 (H)
(D) AT5G67340 ARM repeat protein (U)

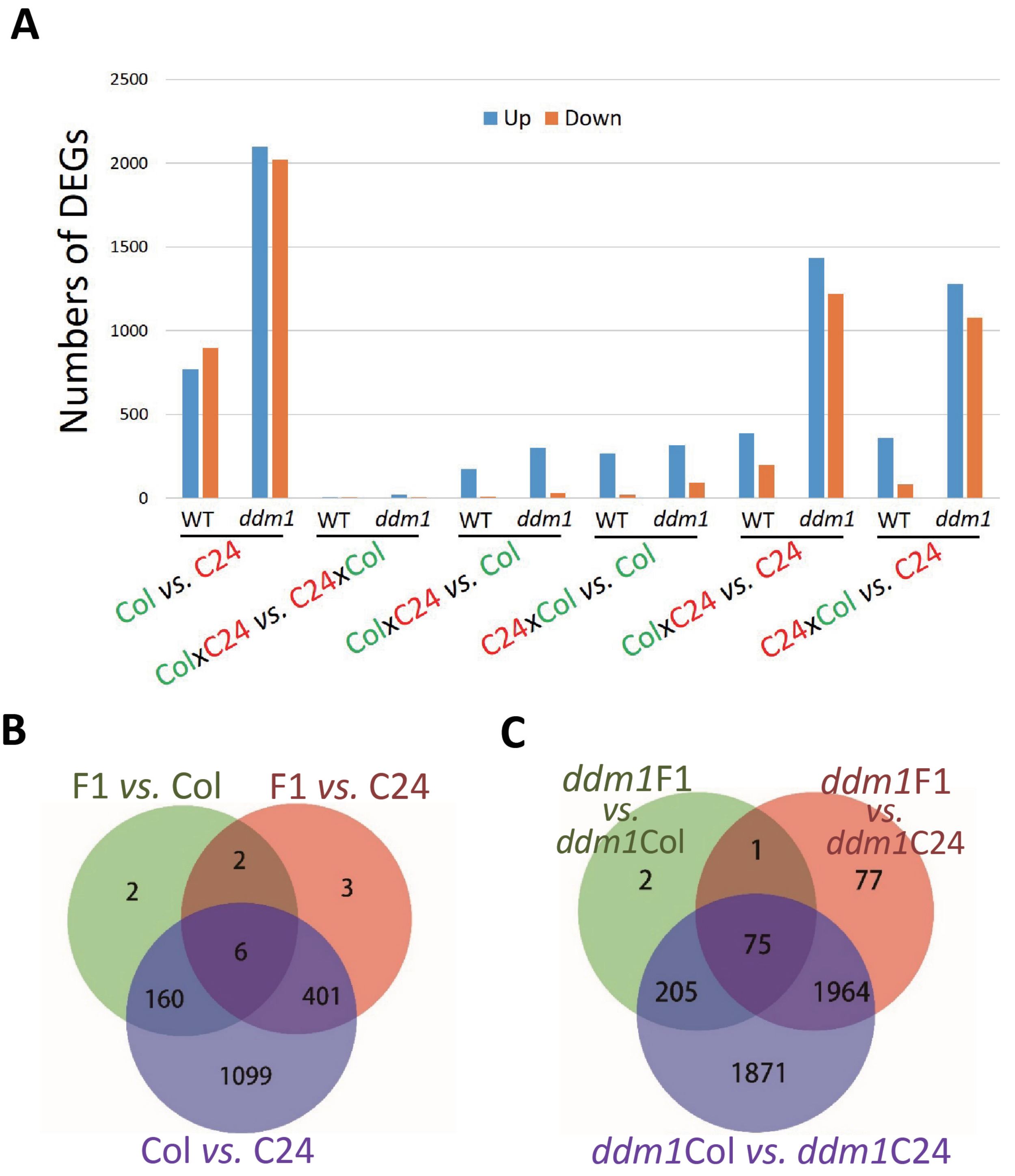
A
Up
Down
Numbers of DEGs
2500
2000
1500
1000
500
0
WT
ddm1
Col vs. C24
ColxC24 vs. C24xCol
ColxC24 vs. Col
C24xCol vs. Col
ColxC24 vs. C24
C24xCol vs. C24
B
F1 vs. Col
F1 vs. C24
Col vs. C24
2
2
3
6
160
401
1099
C
ddm1F1 vs. ddm1Col
ddm1F1 vs. ddm1C24
ddm1Col vs. ddm1C24
2
1
77
75
205
1964
1871

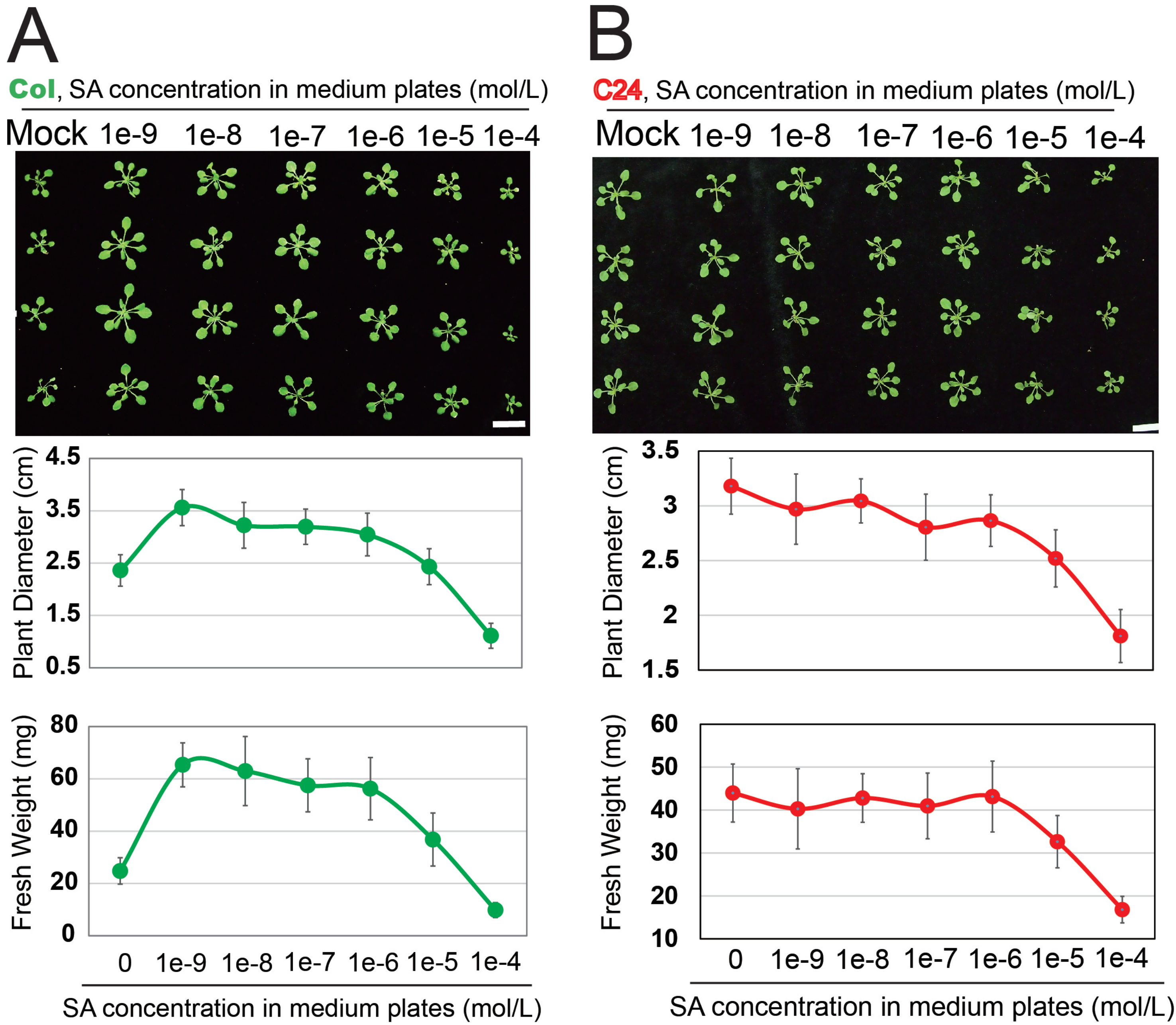
A
Col, SA concentration in medium plates (mol/L)
Mock 1e-9 1e-8 1e-7 1e-6 1e-5 1e-4
Plant Diameter (cm)
4.5
3.5
2.5
1.5
0.5
Fresh Weight (mg)
80
60
40
20
0
0 1e-9 1e-8 1e-7 1e-6 1e-5 1e-4
SA concentration in medium plates (mol/L)
B
C24, SA concentration in medium plates (mol/L)
Mock 1e-9 1e-8 1e-7 1e-6 1e-5 1e-4
Plant Diameter (cm)
3.5
3
2.5
2
1.5
Fresh Weight (mg)
60
50
40
30
20
10
0 1e-9 1e-8 1e-7 1e-6 1e-5 1e-4
SA concentration in medium plates (mol/L)

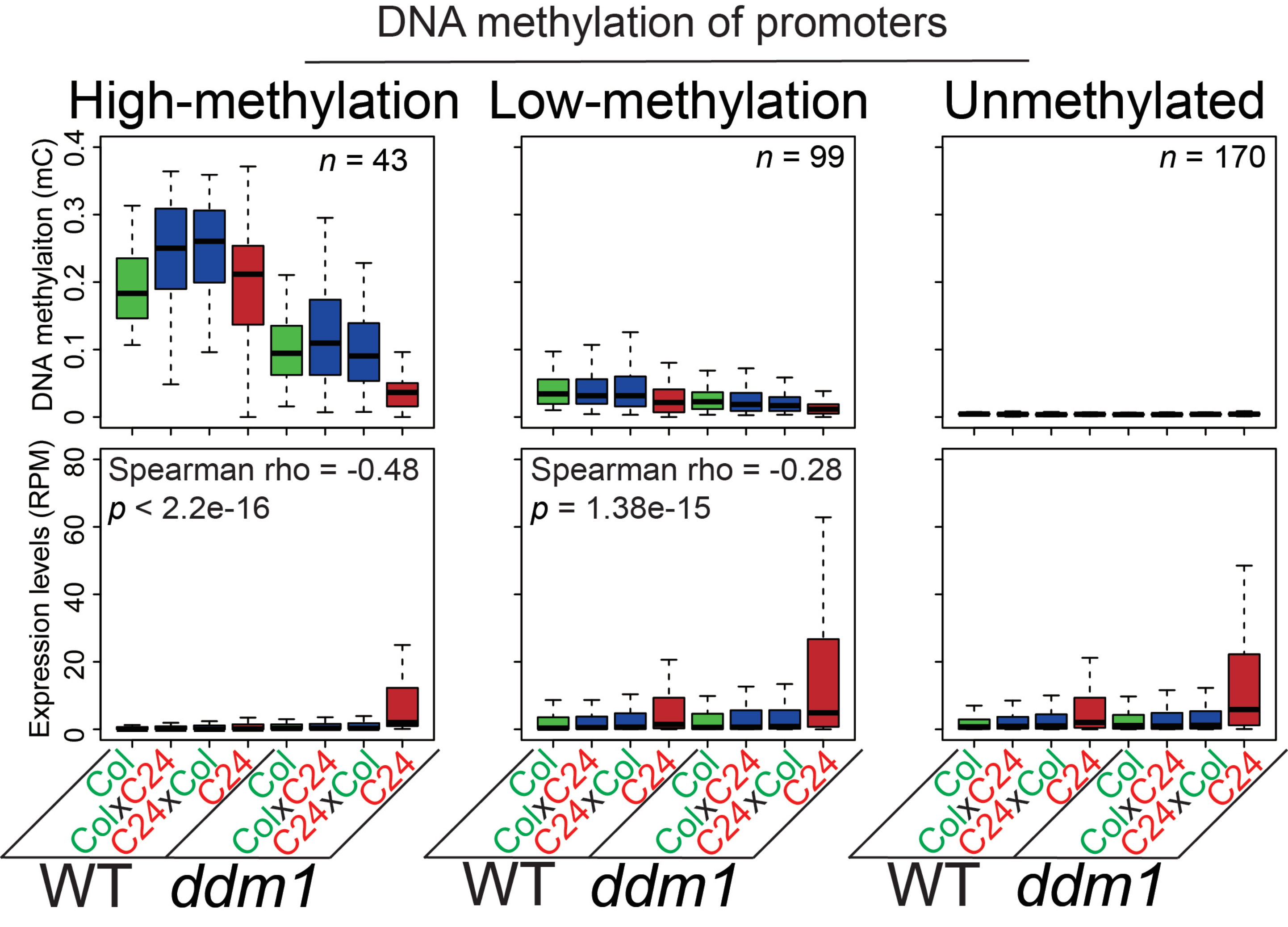

DNA methylation of promoters
High-methylation
Low-methylation
Unmethylated
DNA methylaiton (mC)
n = 43
n = 99
n = 170
Expression levels (RPM)
Spearman rho = -0.48
p < 2.2e-16
Spearman rho = -0.28
p = 1.38e-15
Col
ColxC24
C24xCol
C24
WT
ddm1

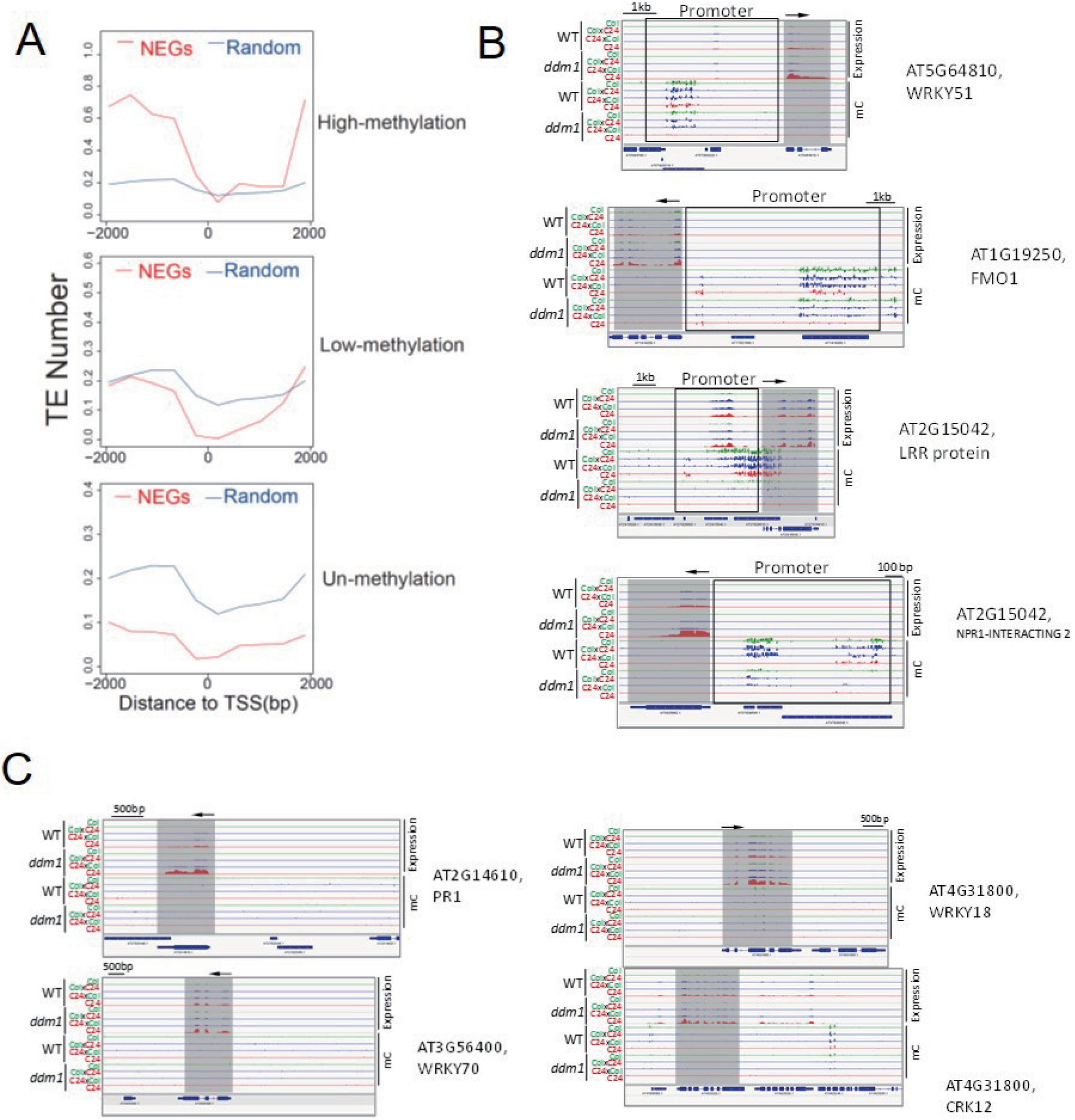

A
NEGs
Random
High-methylation
Low-methylation
Un-methylation
TE Number
Distance to TSS(bp)
-2000
0
2000
B
Promoter
1kb
100 bp
WT
ddm1
Col
ColxC24
C24xCol
C24
Expression
mC
AT5G64810, WRKY51
AT1G19250, FMO1
AT2G15042, LRR protein
AT2G15042, NPR1-INTERACTING 2
C
500bp
AT2G14610, PR1
AT3G56400, WRKY70
AT4G31800, WRKY18
AT4G31800, CRK12
